# Navigating ecological novelty towards planetary stewardship: challenges and opportunities in biodiversity dynamics in a transforming biosphere

**DOI:** 10.1098/rstb.2023.0008

**Published:** 2024-05-27

**Authors:** Jens-Christian Svenning, Melodie A. McGeoch, Signe Normand, Alejandro Ordonez, Felix Riede

**Affiliations:** ^1^ Center for Ecological Dynamics in a Novel Biosphere (ECONOVO), Department of Biology, Aarhus University, Ny Munkegade 114, DK-8000 Aarhus C, Denmark; ^2^ Center for Sustainable Landscapes under Global Change (SustainScapes), Department of Biology, Aarhus University, Ny Munkegade 114, DK-8000 Aarhus C, Denmark; ^3^ Center for Landscape Research in Sustainable Agricultural Futures (Land-CRAFT), Aarhus University, Ny Munkegade 114, DK-8000 Aarhus C, Denmark; ^4^ School of Biological Sciences, Monash University, Clayton, 3800 Victoria, Australia; ^5^ Department of Archaeology and Heritage Studies, Aarhus University, Moesgård Allé 20, 8270 Højbjerg, Denmark

**Keywords:** Anthropocene, biodiversity, climate change, cultural diversity, novel ecosystems, planetary stewardship

## Abstract

Human-induced global changes, including anthropogenic climate change, biotic globalization, trophic downgrading and pervasive land-use intensification, are transforming Earth's biosphere, placing biodiversity and ecosystems at the forefront of unprecedented challenges. The Anthropocene, characterized by the importance of *Homo sapiens* in shaping the Earth system, necessitates a re-evaluation of our understanding and stewardship of ecosystems. This theme issue delves into the multifaceted challenges posed by the ongoing ecological planetary transformation and explores potential solutions across four key subthemes. Firstly, it investigates the functioning and stewardship of emerging novel ecosystems, emphasizing the urgent need to comprehend the dynamics of ecosystems under uncharted conditions. The second subtheme focuses on biodiversity projections under global change, recognizing the necessity of predicting ecological shifts in the Anthropocene. Importantly, the inherent uncertainties and the complexity of ecological responses to environmental stressors pose challenges for societal responses and for accurate projections of ecological change. The RAD framework (resist-accept-direct) is highlighted as a flexible yet nuanced decision-making tool that recognizes the need for adaptive approaches, providing insights for directing and adapting to Anthropocene dynamics while minimizing negative impacts. The imperative to extend our temporal perspective beyond 2100 is emphasized, given the irreversible changes already set in motion. Advancing methods to study ecosystem dynamics under rising biosphere novelty is the subject of the third subtheme. The fourth subtheme emphasizes the importance of integrating human perspectives into understanding, forecasting and managing novel ecosystems. Cultural diversity and biological diversity are intertwined, and the evolving relationship between humans and ecosystems offers lessons for future stewardship. Achieving planetary stewardship in the Anthropocene demands collaboration across scales and integration of ecological and societal perspectives, scalable approaches fit to changing, novel ecological conditions, as well as cultural innovation.

This article is part of the theme issue ‘Ecological novelty and planetary stewardship: biodiversity dynamics in a transforming biosphere’.

## 1. Introduction

Biodiversity is what makes Earth not only habitable but, indeed, a wonderful place to live. Worryingly, however, human activities drive fundamental changes to the biosphere via anthropogenic climate change, biotic globalization, trophic downgrading, and pervasive land use intensification and degradation (e.g. [1–3]). These are of such magnitude that our species *Homo sapiens* has become a major force in the Earth system. This situation is encapsulated in the call to define a new human-shaped geological epoch for our time, the Anthropocene [4], widely suggested to have started by the mid-twentieth century [5]. This ecological planetary transformation with climatic, environmental and biotic conditions shifting into unchartered grounds comes with large risks and may encompass changes spanning gradual, moderate ecosystem shifts to large-scale ecosystem breakdowns, mass extinction and catastrophic climate dynamics (e.g. [6,7]). The concept of novel ecosystems, self-maintaining or, more realistically, self-developing ecosystems without historical precedent owing to human impacts, becomes central in this situation [8,9]. However, their functioning and dynamics remain inadequately understood, as highlighted by Ordonez & Gill [10] in this theme issue, leading to scientific ambiguity and controversies around conceptualizing novel ecosystems [8]. More generally, many aspects of global change-induced ecological dynamics remain incompletely understood and difficult to forecast, and effective mitigation actions are poorly developed and implemented.

The newly published Intergovernmental Panel on Climate Change Sixth Assessment Report puts increased emphasis on the urgency to establish sufficient climate actions to keep the increasingly difficult goal of a maximum 1.5–2°C global temperature above the pre-industrial climate-normal, with ecosystem restoration highlighted as a major potential contributor to climate change mitigation. If not mitigated, however, intensifying human activities and massive climate change could jointly lead to numerous extinctions and the collapse of ecosystems, which in turn may exacerbate climate change and put further pressure on human populations. To avoid such a ‘ghastly future' [11], there is an urgent need for effective planetary stewardship and rethinking what such stewardship could look like (e.g. [7]). Reflecting the urgency of climate and biodiversity crises and the need for sustainable development—all linked to the ongoing planetary ecological transformation—there is rising scientific and political interest in finding solutions [12]. This comes with a demand on science: to produce the knowledge needed for a safer, more sustainable biosphere pathway [13]. It is of the utmost importance to improve our predictive understanding of biodiversity and ecosystem dynamics under human-induced global change and to improve the scientific basis for the necessary transformational change in the human-biosphere relationship. As an example, the current United Nations Decade on Ecosystem Restoration sees a strong and urgent need to better understand current and future biodiversity and ecosystem dynamics in response to restoration actions to properly guide efforts towards long-term success (e.g. [14,15]). Similarly, the recently negotiated Kunming-Montreal Global Biodiversity Framework sets new international conservation and restoration goals for the coming decades [16], which again requires a better understanding of biodiversity and ecosystem responses to various actions amidst rising global change. An integrated approach to governance that considers the intersections across society, policy and information systems has been identified by the Intergovernmental Science-Policy Platform on Biodiversity and Ecosystem Services as necessary to bring about the required progress to address biological invasions [17]. Together, these illustrate the vital need to achieve and integrate a better understanding of likely future biodiversity dynamics to help shape realistic and efficacious policies.

**Figure 1.**
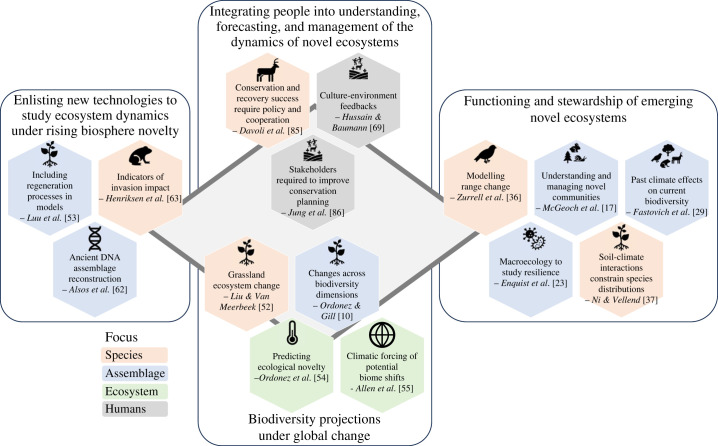
The future of biodiversity and ecosystem dynamics and effective and sustainable stewardship requires knowledge integrated across disciplines and perspectives. Perspectives and examples of how this may be achieved are brought together in this theme issue and its four themes.

In this theme issue, we contribute to tackling this challenge by synthesizing and providing new insights into future biodiversity and ecosystem dynamics. We also explore approaches to future management and sustainable stewardship, covering a range of disciplines and perspectives. The contributions are linked to four subthemes: (i) functioning and stewardship of emerging novel ecosystems; (ii) biodiversity projections under global change; (iii) enlisting new technologies to study ecosystem dynamics under rising biosphere novelty; and (iv) integrating people into understanding, forecasting and management of the dynamics of novel ecosystems (figure 1). Cross-cutting topics addressed include the importance of long-term perspectives, looking beyond the year 2100, and coupling long-term palaeoecological and archaeological perspectives to inform this ‘deep future' perspective; the focus on cross-scale interactions, i.e. how local dynamics and actions will modulate larger-scale forcings and vice versa; and the need for improved integration of the role of people into ecological dynamics.

## 2. Functioning and stewardship of emerging novel ecosystems

Rising environmental and ecological novelty poses strong challenges for responding to current dynamics in ecosystems and biodiversity as well as for forecasting their future dynamics. For instance, the risk of climate-driven dieback of large parts of the Amazon forest remains poorly understood despite its probable disastrous consequences for biodiversity and the climate system at regional to global scales (e.g. [18]). More generally, we have a poor understanding of how tropical ecosystems are likely to develop under a level of warming that is unprecedented for millions of years, e.g. what kinds of vegetation might develop and what would their functioning be? Our predictive challenges come not only from unprecedented warming and CO_2_ levels, but also from the forecast of continuous changes in climatic conditions and the unprecedented biotic mixing owing to globalization (e.g. [19]). Past human land-use has also left important imprints on tropical ecosystems, including the Amazon forest [20], further complicating the task of predicting ecological changes. An important way to improve predictive capacity may come from biological theory such as community assembly, trait-based and metabolic scaling theories (e.g. [21]). Established general relationships between organism size, metabolism, traits and climate offer the potential to forecast states of vegetation (e.g. [22]) and ecosystems under future novel climate conditions. However, high levels of unpredictability—at least at the scale of fine-grained biotic composition—may nevertheless be pervasive. Notably, as Enquist *et al.* [23] highlight in this theme issue, introduced pathogens owing to globalization may cause strong disturbances. Alien tree pests and pathogens are an increasing risk to forest ecosystems where tree species of major importance—foundation species [24]—have already sometimes been lost, leading to major ecosystem disruption, but also often substantial compensatory dynamics (e.g. [25,26]). By this token, such Anthropocene pathogen impacts on forest ecosystems simultaneously illustrates unpredictability in detailed compositional dynamics and predictability regarding broader ecosystem structure and functioning. This case shows that compensatory dynamics may provide some, but not unlimited resilience to anthropogenic disturbance, as discussed by Enquist *et al.* [23]. Hence, there is hope that moderate levels of predictive ability concerning the structure and functioning of future novel ecosystems may be achieved.

Climate change may cause complex dynamics with strong disequilibrium dynamics [27] with long-lasting legacies in both species composition and ecosystem structure and functioning [28]. Such dynamics are likely to contribute to rising ecological novelty and constitute one of the major challenges to our forecasting ability. In this theme issue, Fastovich *et al.* [29] show that reduced temperature change (alongside rising precipitation) at the Pleistocene-Holocene transition helps explain high present-day levels of species richness in mammals, trees, amphibians, reptiles, and, to a lesser extent, birds in the southeast relative to other parts of eastern North America. This study adds further evidence that past climate change may shape diversity patterns across considerable time scales, from thousands to millions of years. Earlier studies have shown such effects on various dimensions of biodiversity, from species richness to endemism and functional diversity (e.g. [30–32]), as well as on ecosystem structure and functioning, e.g. with Quaternary-scale climate instability reducing forest stand productivity [33]. These disequilibrium legacies in biodiversity and ecosystem patterns have been shown to be especially strong in groups characterized by poor dispersal ability [27,31,34]. Their range dynamics are thus strongly dispersal limited, which may cause strong stochasticity in their large-scale dynamics (e.g. [35]). In line with these findings, Fastovich *et al.* [29] report that species richness in eastern North America is less linked to palaeoclimate in birds than in herptiles, trees and mammals. Similarly, Zurell *et al.* [36]—also in this theme issue—show that, in birds, dispersal limitation in response to current climate change is minor within continents, while lagging trailing range edges at the same time suggest widespread extinction debts. Such extinction debts may also contribute to the emergence of non-analogue assemblages and hence ecological novelty, albeit only temporarily. Dispersal limitation may be further exacerbated by additional factors like unsuitable soil conditions at range limits which may reduce the ability of plant species to track ongoing climatic-warming, as reported here by Ni & Vellend [37], losses of plants dispersal agents owing to defaunation dynamics [38], and reduced dispersal agent movement rates owing to habitat fragmentation and other land-use effects [39]. In conclusion, disequilibrium responses to climate change will probably be pivotal in shaping emerging ecological dynamics with a high potential for long-lasting impacts. As these may involve a range of unwanted effects such as increased extinction risks and reduced ecosystem functioning and resilience, careful attention needs to be given to how to manage these dynamics, to the extent this is even feasible, e.g. when to enhance rates of change via assisted migration [40].

Novel ecosystems are widely emerging not just owing to climate change, but also globalization via the human-mediated spread of species across natural dispersal barriers. The latter dynamic is intensifying and is expected to continue doing so in the future [41], already leading to accelerating changes to biotic assemblages [19]. As mixed native and alien species (mixed-species) communities are here to stay in the Anthropocene and are likely to become ever more prevalent [41], a key challenge towards achieving a nature-positive future is how to design the management of mixed-species communities to support positive and avoid negative effects, as discussed here by McGeoch *et al*. [42]. This is an analogous challenge to developing appropriate management responses to the dynamics driven by human-induced climate change [40], which will also require new thinking relative to conventional conservation paradigms, notably adapting to inevitable change and facilitating dynamics towards net positive biodiversity outcomes. In both cases, our understanding of current and future communities can be advanced by considering the six fundamental processes identified for community biology: dispersal, drift, abiotic interactions, within-guild interactions, cross-guild interactions and genetic changes [42,43]. Considering both alien species and species tracking human-induced climate change—neonatives *sensu* Essl *et al.* [44]—decisions on management approaches can be informed by species' movement potential and expected impact in their novel range [42] alongside feasibility considerations. Many alien species have little ecological impact, while some even provide positive impacts. Accordingly, in many contexts, the recommendation is likely to be no action or a low priority for action (e.g. [45]); however, this will not always be the case [17,42]. A new global meta-analysis reports no evidence that large-herbivore impacts on local plant communities depend on nativeness, but rather is shaped by functional traits [46]. This suggests that, at least for some organism groups, traits are more informative than nativeness for the functioning of novel ecosystems. The stewardship of novel mixed-species landscapes of the future will be conditioned by their biodiversity and ecosystem characteristics, how these are aligned to societal values, and the feasibility of potential interventions. Here, McGeoch *et al.* [42] propose that the so-called RAD (resist-accept-direct) framework [47] can be used to guide a more nuanced decision-making under this emerging novelty. Notably, direct (D) approaches accommodate alien species that have neutral or socially desirable ecological effects but include interventions to ‘direct' communities towards more desirable, resilient and biodiverse states, while accept (A) approaches ‘accept' alien-species-caused ecosystem transformations while adapting to reduce any negative impacts. An example of a direct approach would be the restoration of functional, diverse large-herbivore communities—as envisioned in trophic rewilding *sensu* Svenning *et al.* [48]—as a long term, scalable approach to limit the tendency for alien plants to attain high dominance, to the benefit of overall plant diversity including resident native species [49,50].

## 3. Biodiversity projections under global change

Given the ongoing and uncertain nature of expected changes in the Anthropocene era, managing ecological systems requires us to project changes in biodiversity under alternative scenarios, consider the rates of ecological change [40], and take a long-term perspective [51]. Models may be based on individualistic species dynamics [52], be designed as interactive multi-species models [52], focus on the mechanisms of organismal responses [10,53], or take a more holistic assemblage, ecosystem or environmental approach [10,54,55]. Further, projection models can be built either on first principles [23,53,55], statistical associations [36,52] or involve transformations of environmental changes into measures of ecological response [54].

An example of species-based modelling in this issue is the work by Liu & Van Meerbeek [52], which uses species distribution models to predict the impact of climate and land-use changes on grassland communities across Europe. They report that by 2100, grasslands are widely expected to experience strong compositional shifts owing to climate change, with land-use changes also important. Zurell *et al.* [36] also in this issue, also use species-based modelling to show how bird species ranges shifted northward and north-eastward in Europe and westward in North America between 1980 and 2000. As an alternative to these approaches, dynamic vegetation models can be used to simulate the environmental dependencies of specific life stages of a set of plant species and their interactions, not least via competition for light. For instance, in this issue, Luu *et al.* [53] uses this approach to demonstrate the need for incorporating regeneration processes in mechanistic vegetation models to describe changes in composition and ecosystem properties accurately.

Holistic approaches allow a focus on changes in the overall state of a system. For instance, in this theme issue, Allen *et al*. [55] used this approach to assess the global changes in the climate forcing of biome states from now until 2500. Their findings show that under the somewhat optimistic but still possible representative concentration pathway (RCP) 4.5 climate scenario, up to 40% of the terrestrial area is predicted to undergo climatic shifts towards a new biome state, i.e. shifts that will force major ecosystem changes with strong effects on biodiversity and society. Also in this issue, Ordonez *et al.* [54] use a holistic approach to show that under all RCP climate scenarios, over 50% of the biomes are predicted to experience climate changes that could lead to novel ecosystems by 2300. While high levels of change are forecasted to happen before 2100, both studies also estimate the climate-driven changes to continue across the subsequent centuries, highlighting the need, as advocated by Lyon *et al.* [51], to expand our views of change beyond the current end-of-century perspective used in most biodiversity forecasting studies. This is important, not least because there is no reason to think ecological changes induced by global change factors such as climate change and globalization will reach any kind of equilibrium during this century [41,56], given probable longer-lasting change in these drivers, but also the slowness of many environmental and ecological responses [27,56].

**Figure 2.**
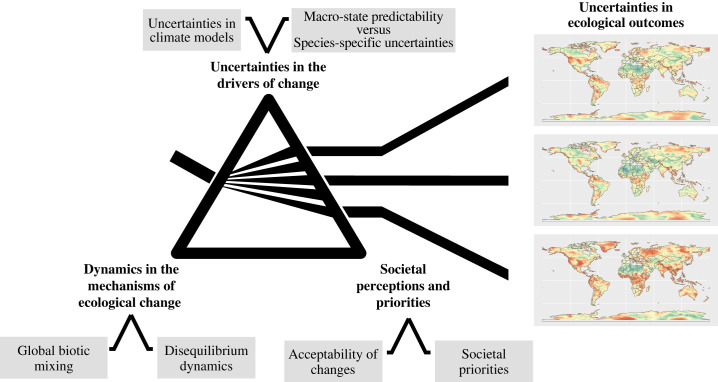
The increasing environmental, ecological and societal changes present significant challenges in predicting the future of ecosystems and biodiversity. The first challenge arises from the diverse potential futures shaped by societal perceptions and priorities, influencing the trajectory of ecological change (depicted here as different scenarios, such as the representative concentration pathways or RCPs). Climate policies, socio-economic developments and land-use priorities define these potential futures; for example, shifts in diet and the allocation of land for animal husbandry or agricultural activities can profoundly impact environmental and ecological dynamics. Furthermore, societies may either embrace the transition to new conditions, adapt to them, or actively resist by taking measures to revert changes and strive for a nature-positive future, as discussed by McGeoch *et al.* [42] in this issue. The second challenge stems from uncertainties in the drivers of change, originating from variability among climate models driving ecological transformations. This variability is visualized here as the range in ecological novelty among models under a given scenario, estimated in [54] in this issue. Additionally, the capacity to model macro-scale ecosystem properties and the inherent uncertainties in species- or assemblage-based perspectives contribute to uncertainties in the drivers of change, as discussed by Enquist *et al.* [23]. The final challenge arises from the dynamics in the mechanisms of ecological change, such as global biotic mixing owing to globalization, impacting interaction networks, and prevailing disequilibrium dynamics.

Projecting ecological trends is a challenging endeavour owing to multiple sources of uncertainty, such as society's perceptions and priorities, uncertainties in the drivers of change, and dynamics in the mechanisms of ecological change (figure 2). Human perceptions and priorities play a pivotal role in shaping the possible future trajectories of ecological change. As discussed in McGeoch *et al.* [42] here, and Roura-Pascual *et al.* [57], societal perception of ongoing ecological transformations determines the actions taken to address them. Variability among climate models introduces uncertainties in quantifying the drivers and pathways of ecological change. Climate policies and socioeconomic developments define possible climatic futures, resulting in alternative RCPs that would mean alternative trajectories of climatic novelty [54] and climatic forcing of biome shifts [55]. Lastly, dynamics in the mechanisms of ecological change, such as biotic mixing owing to globalization and associated changes in biotic network structure [19], defaunation [38,58], mismatches in the dynamics of different dimensions of biodiversity [10], prevalent disequilibrium dynamics [27,59], as well as mis-specified spatio-temporal contexts for change assessment [8,60] generate challenges for projecting ecological changes. Limited empirical data and discrepancies among ecological models add to the complexity of projecting ecological trends, as these hinder precise predictions and the development of universally effective mitigation strategies [23]. The inherent uncertainties surrounding ongoing environmental changes and their ecological consequences underscore the challenges of predicting their full range of impacts and the design of appropriate responses. This highlights the need for a proactive and flexible approach to ecosystem management and conservation planning [40,47] in the face of the accelerating emergence of novel biosphere conditions.

## 4. Enlisting new methods to study ecosystem dynamics under rising biosphere novelty

Advances in global change research are delivering increasing appreciation for the complexity and context-specificity of biodiversity and ecosystem responses. This complexity calls for increasingly sophisticated methods and additional data to understand and forecast the nature and consequences of global change, a call that is being met with rapid advances in technology, data availability and modelling approaches, along with the recognition that a combination of data, multiple methods and approaches yields the strongest inference [61]. For instance, in this theme issue, Alsos *et al.* [62] outlines the potential contribution of ancient sedimentary DNA (sedaDNA) to improve our ability to forecast the composition and functioning of emerging novel ecosystems by identifying the patterns and mechanisms involved in biodiversity and ecosystem responses to past climate change. The authors highlight how sedaDNA analyses can provide valuable insights into the processes that drive ecosystem dynamics under changing environmental conditions by identifying the patterns and mechanisms associated with biodiversity and ecosystem responses to past climate change. Further, the authors propose a combined approach using palaeo-time series, process-based models, and inverse modelling to reconstruct past ecosystem dynamics and inform future ecological forecasting effectively. Overall, such methodological advances also offer promise to improve our ability to forecast the composition and functioning of emerging novel ecosystems.

Species responses to climate change may be complex and varied. Tackling this complexity, Zurell *et al.* [36], in this theme issue, examines changes in range and niche dynamics in European and North American breeding birds by combining kernel density estimation, a null model approach and phylogenetic regression to test a suite of hypotheses. This combination of approaches shows clear evidence for transient dynamics, notably strong lags at trailing edges. In another contribution to the issue, drawing on developments in species data collations, informatics and classification, using open data, repeatable workflows and ecological niche modelling across global climatically suitable areas of multiple invasive alien amphibians, Henriksen *et al.* [63] demonstrate how this information can be integrated and explicitly linked to policy targets on biological invasion. As the process of species redistribution gathers speed [19,41], it will become increasingly necessary to move towards automated workflows for mapping spatial and temporal variation in species and assemblage distributions as a basis for understanding the functional consequences of new species additions to local assemblages. Interpreting the outputs while quantifying data adequacy, Henriksen *et al.* [63] show worldwide growth in the potential impacts of predatory and disease-transmitting invasive alien amphibians.

Several other contributions to the theme issue focus on the importance of representing relatively complex ecological mechanism to achieve adequate representing of species range dynamics. In a fundamental demonstration of the importance of environmental filtering mediating plant species' responses to climate change, Ni & Vellend [37] here demonstrate the role of soil properties in mediating plant species' distribution responses to climate and that soil properties are likely to constrain plant migration. In addition to the assessment of non-climatic environmental factors as potential constraints or facilitators influencing species responses to climate change, in another contribution to this theme issue, Luu *et al.* [53] provide results that underscore the potentially critical significance of incorporating comprehensive demographic processes into forecasts of biotic assemblage and ecosystem dynamics. Specifically, combining a dynamic vegetation forest gap model with seed production and seedling survival as functions of weather and biotic conditions demonstrates that adding these regeneration processes improves the prediction of tree stand species composition, suggesting the need for their inclusion to better forecast future forest dynamics.

Each of these studies points to the need to include and integrate multiple biologically relevant variables and processes into new and advanced methods to measure and forecast biodiversity change, not least in the context of emerging novel biosphere conditions. However, these methodological improvements must be coupled with clear conceptual underpinnings for our understanding of change. This can be achieved by defining change in the right spatio-temporal contexts, as suggested by Kerr *et al.* [8] and Lemoine & Svenning [60]. Additionally, we should understand the links between changes across biodiversity dimensions, as recommended by Ordonez & Gill [10] in this theme issue, and clearly communicate the uncertainties associated the models used for forecasting (cf. [23]). More mechanistic and process-based information will be increasingly important to capture biodiversity dynamics and inform stewardship as the biosphere transforms, not least given the likely rising pervasiveness of ecological and environmental novelty and the probable rising importance of disequilibrium dynamics.

## 5. Integrating people into understanding, forecasting and management of the dynamics of novel ecosystems

Improving our knowledge of biodiversity is vital to efficacious mitigation, conservation and restoration. However, the commitment and resources invested into whatever practises are implemented largely depend on how biodiversity in general and individual species in particular are valued at local, regional, national and supra-national levels. While academic traditions have developed such that different disciplines commonly address biological and cultural evolution, it is now clear that humans have extensively shaped ecosystems across all scales for greater than 10 000 years [7,64,65]. It is the evolved behavioural—that is, cultural—flexibility that has allowed humans not only to expand into virtually all habitats [66] but also to modify these to their own benefits [67] in ways that scale up to have transformative effects on the biosphere [68]. Importantly, not all of these niche construction modifications are or ever have been intended, nor are they all beneficial in the long term. Human-induced megafauna extinctions are a paradigm example of such a modification that was probably largely unintentional, no doubt had immediate pay-offs, but now—many millennia later—carries substantial burdens such as down-graded biotic assemblage structure (e.g. [65]).

Not all the ways in which humans interact with biodiversity are as crass and terminal as extinction. As Hussain & Baumann [69] demonstrate, human modifications of ecosystems reach far back into the Pleistocene (e.g. [70–73]). Across these vast timescales, such interactions have taken many forms with both positive and negative outcomes for humans and their cohabitants in the biosphere. Generally, the frequency and pace of these interactions have increased over time, as has their intensity. Encapsulated in the ‘Palaeoanthropocene' notion [74], these dynamics represent the prelude to our current predicaments and offer future lessons. As here argued by Hussain & Baumann [69], safeguarding cultural diversity and accepting different forms of conviviality (e.g. [70–73]) may be one key strategy to working with novel ecosystems in line with the ‘accept' and 'direct' options within the RAD framework. Cultural diversity and biological diversity often go hand in hand [75,76]. In this context, it is important to note that while global changes and the rise of ecological novelty often herald concerns of biodiversity loss and negative social consequences, there are also positive dynamics, e.g. a growing global commitment to biodiversity restoration (e.g. [16]). Reflecting such societal dynamics, certain megafauna populations are showing continental recoveries [77], even within highly populated areas [78], although setback risks are clear and present [79]. Rural-to-urban migration and associated land abandonment have given rise to new spaces conducive to rewilding [80,81]. Shifts towards more sustainable plant-focused human diets may promote further large reductions in the need for crop- and pastureland, also providing major climate benefits [82]. Further, novel cultural practises illustrate the potential for cultural innovation to foster conviviality, e.g. with specialized food provisioning are supporting one of the world's largest raptor gatherings [83]. Overall, these developments often present trade-offs between ecological and cultural needs, yet they also pave the way for fostering biodiversity under shifting climate patterns and other anthropogenic pressures. Predicting these trends poses a significant challenge because of to intricate socio-ecological interactions and inherent uncertainties. Nonetheless, these perspectives on societal dynamics, cultural diversity and cultural innovation suggest promising avenues for enhancing biodiversity in the Anthropocene epoch and the ongoing planetary emergence of novel ecosystems. At the same time, long-term patterns of cultural evolution make it clear that such paths will be challenging to realize [84].

As shown by Davoli & Svenning [85] in this theme issue, future climate-driven range changes for large herbivores in Europe may push many species into areas where they would be newcomers relative to resident populations of other species, including humans. While the analysis reported by Davoli & Svenning [85] does leave room for a silver lining of increased opportunities for eco-tourism and wildlife-related employment, it paints a fairly bleak picture, especially for the bigger species and those living in high latitudes and under the ever-more likely more severe climate change scenarios. Range shifts are a vital response of large herbivores and other organisms to climate change pressures, as habitat changes driven by climate shifts do not respect national borders. Allowing such range shifts across borders would boost these species' resilience, but present a major environmental governance hurdle, as discussed by Davoli & Svenning [85]. While not modelling such barriers explicitly, their argument is strongly supported by the bibliometric study of Jung *et al.* [86], also in this theme issue. Here, the authors deploy text-mining approaches to scan scientific and policy documents pertinent to European conservation planning. These documents show a prioritization of local perspectives despite the need for integration across scales, an overwhelming lack of stakeholder engagement, and an absence of perspectives that include both past biodiversity states and future scenarios. Encouragingly, Jung *et al.* [86] also point out a number of ways in which current practise may be improved. Many of these (e.g. dispersal corridors) align well with the perspectives offered by other authors in this theme issue (e.g. [42,85]). That said, Jung *et al.* [86] also urge conservation scientists to be more mindful of how their work may feed into policy and decision-making, and the need for stakeholder engagement to make this happen.

## 6. Conclusion

The findings presented in this theme issue highlight many challenges but also a range of actionable insights for navigating the challenges posed by human-induced global changes in Earth's biosphere. As we confront the ever more urgent realities of the Anthropocene, several concrete conclusions emerge. The emergence of novel ecosystems, driven by factors such as climate change and globalization, presents a major challenge to our ability to predict and manage future ecological dynamics (see Kerr *et al.* [8] for a general discussion). As illustrated by contributions to the first subtheme of this theme issue, phenomena such as disequilibrium responses to climate change and globalization-driven pathogen introductions drive complex ecological dynamics and challenge predictive models [23,29,36]. Further, these Anthropocene dynamics have the potential for long-lasting impacts on species composition, ecosystem structure and functioning, with the risk of increased extinction rates, reduced ecosystem resilience and loss of critical ecosystem functions. Hence, the imperative to understand and manage the globally emerging novel ecosystems in the face of unprecedented warming is a critical directive. The uncertainties surrounding the responses of these ecosystems underscore the need for targeted research and stewardship efforts, with a major focus on developing strategies to mitigate the potentially disastrous consequences of climate-driven shifts. While detailed predictive accuracy is likely to remain elusive given the complexity and stochasticity of ecological systems, theory-based approaches may be able to offer coarse-grained predictive power, e.g. owing to compensatory dynamics, as discussed by Enquist *et al.* [23]. Still, given the high levels of uncertainty and change, it is also clear that policy and management responses will need to be adaptable and flexible. The fundamental challenge of bridging scientific insights and actual implementations at scales from individual to super-national stands clear. Scientific knowledge does not in and of itself translate into practise. Hence, mapping and bridging the contextually varied and inevitably value-laden middle ground between these worlds is a priority, as exemplified in the contribution by Jung *et al.* [86].

To address these challenges, we must embrace innovative approaches to ecosystem stewardship—such as the novel ecosystem perspective [9], rewilding approaches [48,87], assisted migration [88], as well as diverse cultural practises [75,76]—adapting to change and promoting biodiversity outcomes, all while aligning with societal values, ethics and feasibility concerns. Key characteristics of adequate approaches are that they can be upscaled to be effective at the large scales needed for effective contributions to planetary stewardship, effective under rapidly changing, novel environmental conditions and societally sustainable. Notably, ecosystem stewardship can only be achieved with people, rather than without or against people. The RAD framework [47] provides a valuable tool for decision-making, allowing us to direct climate- and globalization-induced dynamics towards desirable ecological effects and adapt to transformations while minimizing negative impacts, as discussed here in the context of biological invasions [42]. Yet, vitally, its implementation will have to vary according to cultural and political context. At the same time, it is important to consider that there are ethical arguments for animals and plants being accounted for beyond their ecosystem functions and associated importance for society. Arguably, theirs is a place in the world that is independent of the ecosystem services they perform [89,90]. Clearly, implementing the RAD approach across scales from local to supra-national poses practical, conceptual and ethical challenges. Nonetheless, it offers valuable signposts on our path forward in the face of rising ecological novelty in the Anthropocene.

From the second subtheme, on biodiversity projections under global change, it is clear that the call to extend our temporal perspective beyond 2100 is not just a theoretical consideration but a practical necessity. The analyses in Ordonez *et al*. and Allen *et al*., both in this theme issue, show that while high levels of climatic novelty and climatic forcing of biome shifts can be expected to become widespread even under moderate climate scenarios already before 2100, these dynamics are likely to continue in the following centuries [54,55]. Importantly, dynamics in the direction of such trajectories are already emerging [59,91,92]. Acknowledging the irreversible changes already set in motion calls for a paradigm shift in conservation planning, moving beyond short-term projections to embrace a more comprehensive understanding of the consequences of ongoing environmental changes. This paradigm shift can only happen hand-in-hand with a similar reorientation of political priorities at appropriate scales. The third subtheme emphasizing advancements in methods to study ecosystem dynamics points to avenues for enhancing predictive ability. The emphasis on mechanistic and process-based information underscores the importance of refining our methodologies to capture the complexity of biodiversity and ecosystem responses under rapidly changing, increasing novel conditions.

Integrating human perspectives into the understanding and management of novel ecosystems is likewise not just a theoretical consideration but a practical one. This is clear from the final subtheme of the theme issue. Multiple contributions show how diverse cultural practises and societal decisions may either facilitate or limit conviviality, i.e. the coexistence of humans and the rest of biodiversity [69,85]. The RAD framework's focus on adaptive approaches aligned with societal values may here provide a facilitatory pathway for effective ecosystem stewardship at local to planetary scales in the Anthropocene [47]. That said, it is improbable that the pathway towards such stewardship will be free of conflict as finding the optimal solutions for all stakeholders probably involves friction, compromise and costs. A biodiverse future is unlikely to be free of such complexities.

In summary, the conclusions drawn from this theme issue underscore the need for targeted action to understand and better manage emerging novel ecosystems, extending our temporal perspective in biodiversity forecasting, advancing methodologies to study ecosystem dynamics, and integrating human perspectives through practical decision-making frameworks. Importantly, there will be a rising need for adaptive, flexible approaches that are scalable and efficient under changing, novel ecological conditions. A key requisite here will be ongoing cultural innovation to facilitate conviviality under Anthropocene conditions, alongside the general need for continual cultural adaptation to this epoch's changing environments (e.g. [93]). These conclusions provide a roadmap for researchers, policymakers and conservation practitioners to address the immediate challenges posed by global changes and work towards a more sustainable and resilient biosphere.

## Data Availability

This article has no additional data.
